# Evolution and Emergence of Antibiotic Resistance in Given Ecosystems: Possible Strategies for Addressing the Challenge of Antibiotic Resistance

**DOI:** 10.3390/antibiotics12010028

**Published:** 2022-12-24

**Authors:** Ramganesh Selvarajan, Chinedu Obize, Timothy Sibanda, Akebe Luther King Abia, Haijun Long

**Affiliations:** 1 Laboratory of Extraterrestrial Ocean Systems (LEOS), Institute of Deep-Sea Science and Engineering, Chinese Academy of Sciences, Sanya 572000, China; 2 Centre d’étude de la Forêt, Institut de Biologie Intégrative et des Systèmes, Université Laval, Québec City, QC G1V 0A6, Canada; 3 School of Molecular and Cell Biology, Faculty of Science, University of the Witwatersrand, Johannesburg 2050, South Africa; 4 Department of Microbiology, Venda University, Thohoyando 1950, South Africa; 5 Environmental Research Foundation, Westville 3630, South Africa

**Keywords:** antimicrobial-resistant bacteria, antimicrobial resistance genes, public health, environmental health, horizontal gene transfer, One Health, mitigating strategies, resistome, genomics, evolution

## Abstract

Antibiotics were once considered the magic bullet for all human infections. However, their success was short-lived, and today, microorganisms have become resistant to almost all known antimicrobials. The most recent decade of the 20th and the beginning of the 21st century have witnessed the emergence and spread of antibiotic resistance (ABR) in different pathogenic microorganisms worldwide. Therefore, this narrative review examined the history of antibiotics and the ecological roles of antibiotics, and their resistance. The evolution of bacterial antibiotic resistance in different environments, including aquatic and terrestrial ecosystems, and modern tools used for the identification were addressed. Finally, the review addressed the ecotoxicological impact of antibiotic-resistant bacteria and public health concerns and concluded with possible strategies for addressing the ABR challenge. The information provided in this review will enhance our understanding of ABR and its implications for human, animal, and environmental health. Understanding the environmental dimension will also strengthen the need to prevent pollution as the factors influencing ABR in this setting are more than just antibiotics but involve others like heavy metals and biocides, usually not considered when studying ABR.

## 1. Introduction

The term “antibiotics” refers to the substances naturally produced by microorganisms such as actinomycetes, bacteria or fungi, which can inhibit the growth of other microorganisms and destroy their cells [1]. The introduction of antibiotics into clinical practice was the most incredible clinical breakthrough forward of the 20th century [2]. The introduction of the first antibiotics hugely impacted the treatment of various life-threatening bacterial infections and society by reducing morbidity and mortality [3]. Nonetheless, the most recent decade of the 20th century and the beginning of the 21st century have witnessed the emergence and spread of ABR in different pathogenic bacteria worldwide [4]. Continuous misuse of these valuable compounds has rapidly increased antimicrobial resistance in various pathogens that are effectively untreatable [2]. Thus, some organisms have become resistant to more than one antibiotic simultaneously and have been referred to as multidrug-resistant (MDR); some organisms are even resistant to all known antibiotics and are termed pan-drug resistant [5]. Furthermore, although initially developed to describe *Mycobacterium tuberculosis* strains resistant to first-line of treatment—“resistance to the first-line agents, isoniazid and rifampicin, to a fluoroquinolone and to at least one of the three-second-line parenteral drugs”, the term extremely drug resistance evolved to define any organism resistant to any standard antimicrobial treatment regimen [5]. Although modern scientific technologies have boosted humanity’s hope regarding developing new antibiotics, the current scenario shows few novel antibiotics under development. Simultaneously, antibiotic-resistant bacteria that endure antibiotic treatment are getting increasingly regular, making accessible antibiotics ineffectual. Hence, humanity is confronted by significant adverse public and environmental health impacts. This review examines the history of antibiotics and the ecological roles of antibiotics, and their resistance. In addition, this article adds information on the evolution of bacterial antibiotic resistance in different environments, including aquatic and terrestrial ecosystems, and modern tools used for its identification. Further, the review argues the ecotoxicological impact of antibiotic-resistant bacteria and public health concerns. Finally, it concludes with the possible strategies for addressing the challenge of antibiotic resistance.

## 2. History of Antibiotics 

Since the dawn of time, bacterial infections have had a predominant spot in human diseases [3] and caused death in humans. During ancient times (earlier 1640), Greeks and Indians used molds and other plants to treat wounds and infections, while farmers in Russia used warm soils to cure infected wounds. The doctors from Sumerian and Babylonian used beer soup mixed with turtle shells and snakeskins and a mixture of frog bile and sour milk to treat diseases. Likewise, the Sri Lankan army used oil cake (sweetmeat) as a desiccant and antibacterial agent. Despite the lack of a clear idea about the reason for these illnesses, there were consistent attempts to battle them. 

Microorganisms exist in an unfathomably wide variety. The most prominent microbiologists, including Louis Pasteur (1822–1895) and Robert Koch (1843–1910), strongly believed that microbes must develop lethal weapons (“antibiosis”) to combat their rivals to thrive in a competitive environment, and that those that go through the competition have developed resistance to their opponents’ weapons. They reasoned that because the soil contains the greatest variety of microorganisms, this is where these mechanisms would be most effective. A scientist named Selman Waksman (1888–1973) coined the term “antibiotic” (meaning “against life”) in 1942. He explained that it is something microorganisms make at low concentrations to kill or inhibit the growth of other organisms. The term was used throughout the subsequent 20 years per the abovementioned specification. Although the term is still in use, it has expanded to include the many semi- and fully-synthetic “antibiotics” developed by the pharmaceutical industry.

Rudolph Emmerich and Oscar Löw, two German researchers, created the first antibiotic, pyocyanase, in the late 1890s. It was produced by growing the bacterium *Pseudomonas aeruginosa* in a lab and had questionable efficacy and safety when used to treat cholera and typhus. Later, Salvarsan, an arsenic-based medication discovered by Paul Ehrlich in 1909, was effective against the syphilis-causing bacterium *Treponema pallidum*. In other words, this finding paved the way for future research and development of antimicrobial drugs [6]. Penicillin, derived from the fungus *Penicillium*, was the first antibiotic supplied to doctors in the 1940s. As its development was preceded by years of study and observation during World War II, it is commonly referred to as “a child of the war” [1]. By the late 1940s and early 1950s, antibiotic chemotherapy was well tolerated in clinical medicine after the discovery of streptomycin and tetracycline from *Actinomycetes*. In addition to being efficient against the bacillus causing tuberculosis, these medicines were also effective against other pathogenic bacteria [3]. In this context, the filamentous actinomycetes (64%) were the primary source of most naturally occurring antibiotics, followed by the bacterial and fungal species (Table 1). On the other hand, synthetic derivatives are believed to be efficient against pathogenic microbes. 

First-generation cephalosporins, including parenteral medications like cephalothin (1964) and cefazolin (1970) and oral medications like cephalexin (1967), are the most effective against Gram-positive bacteria, methicillin-susceptible staphylococci, and non-enterococcus streptococci [3]. Unlike first-generation cephalosporins, which are effective against Gram-positive and Gram-negative bacteria, second-generation cephalosporins are more successful in the clinic against Gram-negative bacteria such as *Hemophilus influenzae*, *Enterobacter aerogenes*, and some *Neisseria* spp. [8,9,10]. Further, extended-spectrum cephalosporins such as cefpirome (1983), cefepime (1987), and cefaclidine (1989) have enhanced action against *Enterobacter* spp., *Citrobacter freundii*, *Serratia marcescens*, and severe *P. aeruginosa* infections [11,12,13]. Antibiotics gradually established themselves as life-saving medications. In the middle of the 20th century, a large increase in the number of novel antibiotic compounds developed for medical use was observed. Between the years 1935 and 1968, a total of 12 new classes were introduced. However, there was a significant decline in the number of new classes after this; between 1969 and 2003, merely two new classes were developed [14].

## 3. Rise of Antimicrobial Resistance

The term “antimicrobial resistance” (AMR) is used to describe the ability of bacteria and other microorganisms to resist the adverse effects of an antimicrobial to which they were formerly susceptible [15]. Antimicrobial resistance (AMR) was first noted in staphylococci, streptococci, and gonococci; penicillin-resistant *S. aureus* emerged in 1942 following the introduction of penicillin as a commercial antibiotic in 1941 [16]. However, in the early 1930s, Sulphonamide-resistant *Streptococcus pyogenes* appeared in human clinical settings. Later in the 1950s, the problem of multidrug-resistant enteric bacteria became evident [17]. Furthermore, methicillin, which is linked to penicillin and is a semi-synthetic antibiotic, was marketed in 1960 to treat *S. aureus* infections resistant to penicillin. Conversely, in the very same year, methicillin resistance emerged in *S. aureus* [18]. Since their introduction in the 1980s, fluoroquinolones have revolutionized the treatment of bacterial infections. Initially intended for use against Gram-negative bacteria, the emergence of fluoroquinolone resistance has shown that these medications have also been applied to combat Gram-positive infections, most notably among methicillin-resistant strains [19]. Furthermore, although Vancomycin has been on the market for 44 years, in 2002, clinical isolates of Vancomycin-resistant *S. aureus* (VRSA) emerged [20].

A rise in deaths worldwide is attributed to bacteria resistant to multiple antibiotics. For example, there are over 63,000 annual deaths in the United States of America (USA) due to hospital-acquired bacterial infections [21]. Further, in 2019, the Centre for Disease Control (CDC) reported that over 2.8 million antibiotic-resistant infections occurred annually in the United States, leading to over 35,000 deaths [22]. The Indian Council of Medical Research (ICMR) released its annual report on antimicrobial resistance in 2020, which stated that the overall proportion of MRSA throughout the country had reached 42.1% in 2019, representing an increase of nearly 10% compared to the previous year [23]. According to the latest Global Antimicrobial Resistance and Use Surveillance System (GLASS) project report, data from South and Southeast Asian countries (such as India, Bangladesh, and Pakistan) reflect a considerable rise in antibiotic resistance levels. For instance, carbapenem-resistant *Acinetobacter* was found to be exceptionally high in Pakistan (66.9%), followed by India (59.4%). Similarly, the highest prevalence of carbapenem-resistant *E. coli* and carbapenem-resistant *K. pneumonia* was recorded in India (16.4% and 34.2%, respectively), followed by Bangladesh (9.2% and 11.2%) and Pakistan (6.2% and 11.3%) respectively. The other MDR pathogens, such as fluoroquinolone-resistant *Salmonella* sp. (80.3%) and MRSA (65%), were recorded as high in Pakistan [24]. According to the Antimicrobial Resistance Surveillance System (CARSS) and the China Antimicrobial Surveillance Network (CHINET), the antimicrobial resistance profiles of gram-negative bacilli are higher in China. There has been an increase in the incidence of carbapenem-resistant *Klebsiella pneumoniae* since 2005, and the prevalence of extended-spectrum-lactamases and antimicrobial resistance in *Acinetobacter baumannii* are both concerning. Furthermore, the incidence of methicillin-resistant *Staphylococcus aureus* and vancomycin-resistant *Pseudomonas aeruginosa* both declined between 2005 and 2017 [25]. According to a report published by the European Antimicrobial Resistance Surveillance Network (EARS-Net), between 2015 and 2019, there were shifts in the frequency of antimicrobial resistance throughout the European Union. These changes were based on the species of bacteria, with *E. coli* being the most common (44.2%), followed by *S. aureus* (20.6%), *K. pneumoniae* (11.3%), *Enterococcus faecalis* (6.8%), *P. aeruginosa* (5.6%), *Streptococcus pneumoniae* (5.3%), *E. faecium* (4.5%), and *Acinetobacter* spp. (1.7%) [22]. 

Due to limited resources and the difficulty of monitoring medicine supply systems within and outside their borders, many African countries struggle to protect their populations from unsafe and substandard/counterfeit medicines. Several African countries have not yet banned oral artemisinin monotherapies for uncomplicated malaria, for example. This is a major risk for developing resistance to artemisinin-based combination therapies [26]. In all African regions, *S. aureus*, *Klebsiella* sp., *E. coli*, and *S. pneumoniae* exhibited lower resistance to carbapenems and fluoroquinolones than other antibiotic combinations. In West Africa, *Klebsiella* spp. resistance to ciprofloxacin was greater than in other regions [27]. In conclusion, antimicrobial resistance has emerged as a severe threat to human health in the last decades, responsible for an estimated 700,000 annual deaths worldwide; is is anticipated to result in millions of deaths by 2050 if not adequately addressed [28].

## 4. What Caused These Organisms in the Environment to Develop Resistance to Multiple Drugs?

Bacteria are distinct in that they can acquire genes from the parent microorganism during division (vertical gene transfer) and from the larger community (horizontal gene transfer), first demonstrated for aminoglycoside resistance [29]. This horizontal gene transfer has been observed at every major taxonomic rank, even between bacteria and archaea. A strain that was once susceptible may acquire and transfer resistance to a new species or genus. Most antibacterial resistance genes are carried on plasmids (Table 2) and other mobile genetic elements (transposons, genomic islands, integrons, and gene cassettes) that can and do spread to bacteria of different genera and species [28]. Antibiotic resistance due to plasmids is widespread and includes resistance to many first-line treatments. Notable examples include fluoroquinolones, aminoglycosides, and cephalosporins, which are used extensively [30].

Staphylococci isolated from the clinical setting frequently contain multiple plasmids, and this was the first instance of antibiotic-resistant bacteria posing a severe threat to hospital infection control [31]. Plasmids that encode resistance to sulfonamides and other antibiotics have been found in multidrug-resistant *Salmonella enterica* Typhimurium DT104 strains, suggesting that trimethoprim resistance is also encoded in these plasmids [32]. Similarly, vancomycin resistance genes have been found on large plasmids easily transferable in both *E. faecalis* and *E. faecium* [33]. Resistance plasmids in *Enterobacteriaceae* frequently contain narrow-spectrum beta-lactamases (such as penicillinases) and extended-spectrum beta-lactamases (ESBL). Multiple beta-lactamase genes, which can hydrolyse a wide variety of beta-lactam antibiotics, are frequently discovered to be located on the same plasmid [34]. Further, *Enterobacter* spp. harbor plasmids containing the intrinsic *Amp*C-lactamases gene, conferring resistance to ampicillin, amoxicillin-clavulanate, and first- and second-generation cephalosporins [35]. The first instance of plasmid-mediated quinolone resistance (PMQR) was discovered on a *qnr* plasmid in *K. pneumoniae* from a health centre. Subsequently, *qnr* was renamed *qnr*A and families of *qnr* genes (*qnr*B, *qnr*S, *qnr*C, and *qnr*D) due to differences in plasmid copy number and gene expression. Comparing the effects of carriage by highly antibiotic-susceptible laboratory strains frequently reveals the greatest variations [36]. The third group of plasmid fluoroquinolone-resistance genes are the *oqx*AB and *qep*A efflux systems, which code for transporters that can export fluoroquinolone molecules. Yet again, the carriage of these genes is associated with slight increases in the resistance to fluoroquinolones [37].

**Table 2:** Plasmid-borne genes for antibiotic resistance in different organisms.

Antibiotic Resistance	Plasmid-Borne Genes	Resistant Organisms	References
Beta-lactams	*bla*_IMP_ encoding imipenem resistance; *bla*_VIM_ (Verona integron- encoded metallo-β-lactamases)	*P. aeruginosa*	[38,39]
	*bla*_OXA_ encoding oxacillin resistance	*S. aureus*	[40]
	*bla*_NDM_ encoding metallo-β-lactamase	*E. coli*	[41]
	*bla*_NDM-1_ gene; *bla*_OXA_-_23_	*A. baumannii*	[42]
	*bla*_IMP-9_, *bla*_SIM-2_, and *bla*_VIM-2_	*P. aeruginosa*	[42,43]
	*bla*_NDM_, *bla*_IMP_, *bla*_IMP-27_, *bla*_VIM_, and *bla*_KPC_	*Enterobacteriaceae*	[44,45]
	*bla*_NDM-1_	*E. coli*	[46]
	*bla*_IMP_	*Metagenome*	[39]
	*bla*_OXA-23_	*A. baumannii*	[42]
	*bla*_TEM_; *bla*_SHV_, *bla*_OXA_	*H. influenzae*; *E. coli*; *K. pneumoniae*	[47,48]
	*bla*_TEM_	*S. pneumoniae*	[49]
	PBP2a	*S. pneumoniae*; *E. coli*	[50,51]
	CTX-M, OXA-30	*E. coli*	[52]
	*mec*A,	*S. aureus*	[53]
	*bla*_OXA-48_	*Enterobacteriaceae*	[54]
Fluoroquinolones	*qnr*A, *qnr*B, *qnr*C, *qnr*D, *qnr*S, and *aac(6′)-lb-cr*	*Campylobacter* spp., *Salmonella* spp., and *Shigella* sp., *K. pneumoniae*, *E. coli*	[55,56]
	*gyr*A	*E. coli*	[57,58]
	*par*C and *par*E	*E. coli*; *K. pneumoniae*	[57,59]
	*Nor*C, *Nor*A and *Mep*A	*S. aureus*	[60]
	Rv1634	*Mycobacterium tuberculosis*	[61]
	*Mfp*A	*Mycobacterium*	[62]
	*qnr*S2	*Aeromonas*	[63,64]
	*Qep*A	*E. coli*	[65]
	*OqxA*B	*E. coli*	[66]
	*par*C and *gyr*A	*S. pneumoniae*	[67,68]
	Sme_VWX_	*S. maltophilia*	[69]
	Sm*qnr*	*S. maltophilia*	[70]
	Sme_DEF_	*Stenotrophomonas maltophilia*	[71]
	*pqs*A	*P. aeruginosa*	[72]
	*glp*D, *ygf*A, and *yig*B	*E. coli*	[73]
Glycopeptides	*van*A, *van*B, *van*C, *van*D, *van*E, *van*G, *van*L, *van*M, and *van*N	*Enterococci*	[74,75,76]
	*van*R_SHAX_	*S. aureus*	[76]
	*sar*A		[77]
	*van*A and *erm*B	*Enterococci*	[78]
Aminoglycosides	*aac(3)-IV*	*E. coli*	[79]
Polymyxins	*mcr*-1	*E. coli*	[80]
Tetracyclines	*tet* genes	*Streptomyces*	[81]
	Tn916	*B. subtilis*	[82]
	Tet38	*S. aureus*	[83]
Lipopetides	*pit*A	*S. aureus*	[84]
Rifampicin	*pur*B and *pur*M	*S. aureus*	[85]
Cephalosporins	*bla*_CTX-M_, *bla*_CMY_	*Kluyvera ascorbata*; *Kluyvera georgiana*	[86]
	*bla*_CTX-M-1_ and *bla*_CMY-2_	*E. coli*	[87]
*Van*comycin	*van*A, *van*B, *van*H, *van*R, *van*S, *van*W, *van*X, *van*Y, and *van*Z	*S. aureus*	[88]
Multidrug resistance (MDR)	*acr*B	*E. coli*	[89]
	*SGI*1	*S. enterica*	[90]
	*bla*_NDM-1_	*P. aeruginosa*	[91]

Like plasmids, resistance transposons are mobile genetic elements that carry resistance genes. For example, many Gram-negative bacteria, especially *Enterobacteriaceae*, contain the transposable element Tn5 (encodes resistance to aminoglycosides like kanamycin and neomycin) and Tn10 (encodes resistance to tetracycline). Other gene clusters include Tn3, encoding resistance to numerous β-lactam antibiotics, including ampicillin, and Tn21, encoding resistance to streptomycin, spectinomycin, and sulphonamides [30]. A recent study investigated Tn7-like transposons in *Enterobacterales* isolates from food animals. The study found that 54.9% of the isolates were multidrug-resistant, and high resistance rates were observed against streptomycin and trimethoprim-sulfamethoxazole [92].

Resistance integrons are conserved sequences that, through site-specific recombination, can acquire gene cassettes that can transport drug-resistance genes [93]. For instance, *K. pneumonia* and *K. oxytoca* isolates resistant to gentamicin and cotrimoxazole were observed in patients with nosocomial infections. It was found that a significant number of these isolates carried integrons that contained inserted regions of foreign DNA encoding antibiotic resistance genes [94]. Four known classes of resistance integrons to date. Class I and II integrons contain multiple gene cassettes that code for antimicrobial resistance mechanisms like dihydroflavonol-4-reductase (dfr), broad-spectrum-lactamase (bsl), lipoprotein signal peptidase, quaternary ammonium compound (QAC) enzymes, *sul*1 (sulfonamide), and aminoglycoside-modifying enzymes (AMEs) [93]. In addition, these integrons have been observed in Gram-negative organisms such as *Acinetobacter*, *Aeromonas*, *Alcaligenes*, *Burkholderia*, *Campylobacter*, *Citrobacter*, *E. coli Pseudomonas*, *Klebsiella*, and *Salmonella* sp. [95,96,97]. Class III integrons were first discovered in *S. marcescens* transferred by Tn402 in Japan in 1993; however, they are not as active as other classes. However, the IncQ plasmid from *E. coli* has recently been found to contain a class III integron encoding *bla*_GES-1_ (an ESBL-encoding gene) [98,99,100]. In addition, gene cassettes that confer resistance to fosfomycin and chloramphenicol have been identified in class IV integrons [101]. However, current research is limited to class I integron and Gram-negative bacteria. Class I integron on Gram-positive microorganisms, along with classes II, III and IV, has barely been touched, making such concerns unnoticed about antibiotic resistance determinants. Further, the complex origin of antibiotic resistance still hinges on several factors. These include antibiotic overuse and abuse, inaccurate diagnosis, inappropriate antibiotic medicating, loss of responsiveness in patients, patients self-medicating, poor healthcare settings, lack of personal hygiene, and pervasive agricultural use [102,103].

## 5. Mechanisms of Antibiotic Resistance

The development of antibiotic resistance is a natural ecological phenomenon and the product of billions of years of evolution. However, much attention has been focused on antibiotic resistance in pathogenic organisms encountered in hospitalized patients and bacteria responsible for adverse health effects [104]. In addition, microbes in pristine environments, such as caves and permafrost, have been studied and found to develop resistance in the absence of human interference. Antibiotics are used for a wide variety of bacterial infections in humans and animals. This promotes the generation of resistance or “immunity” genes in the producer organisms and the selection of resistance in environmental species. The presence of resistance in the natural environment may be a natural occurrence’ this reservoir of resistance genes can be mobilized and transferred into human pathogens, worse the situation [105,106,107]. The presence of identical genes in both environmental and human bacteria demonstrates the movement of resistance genes from different environmental reservoirs, including aquatic and terrestrial environments, into human pathogens and vice versa. Furthermore, environmental microorganisms already have genes encoding resistance to antibiotics before they are widely used commercially [108,109].

**Figure 1:**
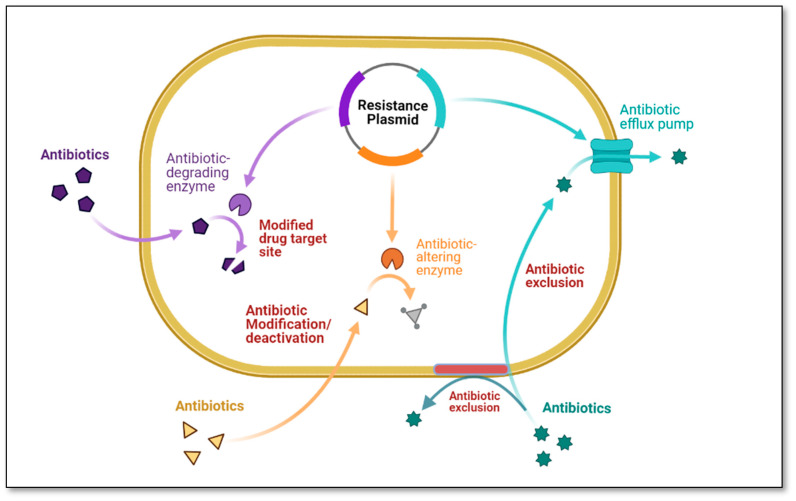
Antibiotic resistance mechanisms (Figure created using Bio-render).

The development of antibiotic resistance has brought to light a plethora of diverse and intricate mechanisms responsible for the genesis and propagation of antibiotic resistance among bacteria of the same species or even among bacteria of different species [110]. Important resistance mechanisms shown in Figure 1 include (i) antibiotic exclusion by the cell membrane, (ii) antibiotic modification and/or deactivation within the cell, (iii) reduced sensitivity of the cellular target, (iv) antibiotic exclusion from the cell, and (v) intracellular sequestration [111]. These multiple processes mediate antibiotic resistance enabling bacteria to become resistant to all currently available antibiotics. For instance, three biochemical pathways can lead to fluoroquinolone resistance; these pathways can exist in the same bacteria at the same time with increased expression and, often, increased resistance levels such as (i) overexpression of efflux pumps that effectively remove the drug from the cell, (ii) mutations in genes that encode the target site of fluoroquinolones (DNA gyrase and topoisomerase IV), and (iii) protection of the fluoroquinolone site of action by a protein named Qnr [112]. 

The most common resistance mechanism in Gram-negative bacteria is the production of beta-lactamases; in Gram-positive organisms, resistance is typically achieved by alteration of the target site, i.e., penicillin-binding proteins (PBPs) [112]. One of the unique mechanisms of antibiotic resistance is the efflux pumps system, which pumps antibiotics and other toxins out of the cell. This mechanism is crucial in bacteria becoming resistant to the antibiotic. On the other hand, Gram-negative bacteria acquire tetracycline resistance via efflux pump systems, specifically the *tet* efflux pumps, which export tetracyclines from cells through proton exchange [113]. Other MDR efflux pumps that extrude tetracycline are AcrAB-TolC and MexAB-OprM, found in *Enterobacteriaceae* and *P. aeruginosa* [114]. Finally, resistance to trimethoprim is caused by alterations in metabolic pathways, such as the increased production of dihydrofolate reductase, an enzyme that lacks the binding site for trimethoprim [115], and dihydropteroate synthase. This enzyme mediates resistance to sulfonamides [116]. Table 3 explains the different mechanisms is mediating antibiotic resistance.

## 6. Antibiotic Resistance in Different Environments

Excessive antibiotic use results in antibiotics being released into different environments, including aquatic and terrestrial environments. The reduced effectiveness of antibiotics against human and animal pathogens is a significant concern raised by the widespread release of antibiotics into these environments. Globally, the public and scientific community are becoming increasingly concerned about antibiotics in the given environment [148].

### 6.1. Aquatic Environments

Freshwater ecosystems are among the natural settings that have the potential to become contaminated with antibiotics released into the environment by a wide range of sources, including agricultural runoffs, sewage discharges, and leaching from nearby farms [148]. Mobile genetic elements (MGEs) encoding antibiotic resistance spread quickly through horizontal gene transfer (HGT) in aquatic environments [149] and have played an essential role in the development and spread of resistant bacteria into the environment, which can cause infections in both humans and animals. Water bodies like rivers, streams, wastewater effluents, and lakes are connected ecological habitats that have received increased scrutiny recently due to evidence that they play a significant role in spreading antibiotic-resistant genes [21].

#### 6.1.1. Wastewater

##### Wastewater Treatment Plants

Wastewater treatment plants (WWTPs) are a repository for numerous organic compounds, nutrients, and metals [150]. Traditional wastewater treatment plants are very effective at removing organic compounds and nutrients from wastewater but are not designed to eliminate antibiotics [151]. It was previously thought that biological or chemical degradation, or sorption to sludge, removed some antibiotics that inevitably made their way to WWTPs from both human and animal usages [152]. Some approximations can be made using hydrophobicity and partitioning coefficients for the propensity of antibiotics to sorb to organic matter in WWTPs [153]. For instance, sulfonamides and trimethoprim are shown to have a lower sorption potential than fluoroquinolones and tetracyclines, but both sorb strongly to solids. As evidence, a study found that tetracycline concentrations across ten Chinese WWTPs were much lower than sulfonamide concentrations [154]. While biodegradation plays another significant role in antibiotic removal during wastewater treatment, it is typically seen to be less important than sorption in the removal of the most common antibiotics studied [153,155]. Despite this, sulfonamides and several beta-lactams [153,156] exhibit low sorption characteristics and have an important removal mechanism through the biodegradation process. Although there are many stages of treatment in place at WWTPs, antibiotics in wastewater are not entirely removed and/or degraded, leading to persistent accumulation. For instance, wastewater treatment facilities have been singled out as a potential origin of HGT, contributing to the development of antibiotic resistance, which in turn increases the concentration and overexpression of antibiotic resistance genes (ARGs) in the wastewater system [157]. 

The effluents from WWTPs contain high concentrations of antibiotic-resistant bacteria (ARB) and ARGs [157], typically found in aquatic environments. For instance, WWTP processes can harbor resistant and MDR bacteria such as *Enterobacteriaceae*, *P. aeruginosa*, *E. coli*, and *Acinetobacter* sp. [158]. However, various clinically significant ARBs, including MRSA, VRE, and a few Gram-negative bacteria, have been identified. These bacteria produced ESBLs and were resistant to fluoroquinolones and carbapenems [159,160]. Martins da Costa et al. [161] studied antimicrobial resistance in *Enterococcus* spp. and reported that biological treatment at WWTPs was ineffective in preventing the spread of MDR enterococci from urban sewage and sludge in Portugal. In addition to resistant bacteria found with culture-dependent methods, genes conferring resistance to all antibiotic classes have been found in WWTPs effluents globally using culture-independent methods. For example, 30 ARGs encoding resistance to tetracycline, sulphonamides, quinolones, and macrolides were found in the activated sludge of two WWTPs in China. Additionally, ten ARGs were significantly increased compared to the abundance of the *16S rRNA* genes [162]. Similarly, a survey of 16 urban WWTPs across ten European countries found a wide range of ARGs in the effluent, including *sul*1, *tet*M, *bla*_OXA-58_, *bla*_TEM,_ *bla*_OXA-48_, *bla*_CTX-M-32_, *mcr*-1, *bla*_CTX-M-15_, and *bla*_KPC-3_ and reported that the majority of ARGs were found in water bodies downstream from WWTP discharge points [163]. 

##### Hospital and Pharmaceutical Wastewater

Since the widespread use of antibiotics in hospitals leads to the excretion of their active forms into the environment, clinical sewage has long been recognized as a significant source of antimicrobial resistance determinants in aquatic environments [164]. Similarly, substantial amounts of antibiotics, and other compounds in pharmaceutical wastewater exert selection pressure even at concentrations well below therapeutic levels [165]. Many recent investigations have focused on pharmaceutical wastewater (PWW) and hospital wastewater (HWW) to examine the resistomes and the associated health risk [166,167,168,169]. For instance, Obayiuwana and Ibekwe reported that PWW exhibited a variety of ARGs, including *cat*A1 (58.3%); *sul*I (31.7%); *tet*E (30%); *aac(3)-IV* (28.3%); *erm*C (20%); *bla*_TEM_, *bla*_CTX-M_, *bla*_NDM-1_ at (18.3% each), encoding resistance to chloramphenicol, sulfonamides, tetracycline, aminoglycoside, macrolide-lincosamide-streptogramin, and β-lactams and penicillins, respectively [170]. Another recent meta-analysis of HWW revealed a similar pattern, with high levels of resistance genes to carbapenems, sulfonamides, tetracyclines, and mobile genetic elements. From 2014 to 2018, there was a significant decline in the number of resistance genes to ESBLs, carbapenems, sulfonamides, and glycopeptides, while there was an increase in the number of genes that were resistant to tetracycline [171]. Many previous studies have highlighted the need to increase wastewater treatment capacity in developing countries, focusing on hospital wastewater. Since untreated wastewater is sometimes used to irrigate crops, this could result in the spread of resistant bacteria to the food supply and the local population. Although this poses no immediate risk to produce, the more prevalent these bacteria are, the more likely AMR might spread, especially among immune-compromised individuals or those undergoing surgery. The global COVID-19 pandemic has recently resulted in a spike in demand for antibiotics. This is because a sizable subset of COVID-19 patients also required antimicrobial therapy for secondary bacterial or fungal infection [172]. This increased the concentrations and diversity of these pharmaceuticals in HWW. Therefore, inadequate disposal of non-metabolized antibiotics into hospital sewage systems is also a source of antibiotic-resistant microbes in the aquatic environment.

#### 6.1.2. Rivers and Groundwater

Rivers are potential compartments where environmental, human, and animal-related bacteria can coexist, at least in the short term, because they receive ARB from various sources, such as WWTPs, urban runoff, and industrial or agricultural activities [173]. In addition, ARGs can be released or spread by ARB and are relatively stable and accessible to other bacteria, resulting in the evolution of a new generation of bacteria resistant to antibiotics [174]. 

Genes for aminoglycoside resistance, such as *aac*, *aph*, and *ant*, are widely dispersed throughout many different genera, including *Aeromonas*, *Escherichia*, *Vibrio*, *Salmonella*, and *Listeria* spp., which have been isolated from river water [175]. Similarly, ARGs encoding resistance to other aminoglycoside group antibiotics, such as phosphotransferase genes encoding resistance to neomycin (*np*tII) and streptothricin (*str*AB), have also been detected in river water in Canada [176] and India [177]. Microorganisms in river water with high concentrations of antibiotics due to urban and agricultural activities were found to carry sulphonamide resistance genes like *sul*I, II, III, and A [178]. However, there is evidence that some rivers have ARGs, including stretches showing no pollution. The four *sul* genes (*sul*I, II, III, and A) found in bacteria isolated from a pristine river suggest that *sul*I, as a component of class I integrons, can be disseminated and transferred horizontally within and between bacterial species in river water [179].

Antibiotics can make their way into the groundwater and even a water supply that people drink *via* surface water [180]. Not surprisingly, antibiotic exposure in groundwater was less than in surface water [181]. However, there is substantial evidence in the scientific literature linking microbial contamination of groundwater to adverse public health outcomes [182,183,184,185]. According to a recent review by Murphy et al. [186], there is strong epidemiological evidence of disease transmission due to groundwater contamination on a global scale, with an annual estimate of 35.2–59.4 million cases of acute gastrointestinal infection potentially attributable to groundwater consumption. The global disease burden is already high, and the potential implications of groundwater-borne ARB are even more alarming.

#### 6.1.3. Marine System

The oceanic ecosystem has received the least attention among the aquatic environments. There is a possibility that antibiotic release is not subject to significant selection in oceans because of the high diffusion rates. However, the presence of AMR in marine environments can potentially be caused by one of three different mechanisms. The first way is transporting ARB from terrestrial sources to coastal environments via runoff. In this scenario, ARGs should be present in bacterial taxa that are generally not found in the ocean. The second mechanism is antimicrobial resistance selection due to anthropogenic antibiotic runoff, which encourages naturally occurring bacteria to become resistant to the antibiotics. The third factor is the development of antibiotic resistance as a direct result of the production of antibiotics in marine environments [187]. Using metagenomic sequencing, a recent baseline study found ARGs in 12 coastal environmental samples from the urban coastline of Kuwait. The authors detected 402 ARGs in these samples, with the most common being *pat*A, *ade*F, *Erm*E, *Erm*F, *Tae*A, *tet*X, *mph*D, *bcr*C, *srm*B, *mtr*D, *bae*S, *Erm*30, *van*TE, *VIM-7*, *Acr*F, *ANT4-*1a, *tet*33, *ade*B, *efm*A, and *rps*L. The beta-lactams (cephalosporins and penam) elicited the highest levels of resistance, and 46% of the genes originated from Proteobacteria. Also, ESKAPEE pathogens (*Enterococcus faecium*, *S. aureus*, *K. pneumonia*, *Acinetobacter baumannii*, *Pseudomonas aeruginosa*, *Enterobacter* sp., and *Escherichia coli*) were found in low concentrations [188]. Nonetheless, future research into MGEs and integrons will be necessary to monitor the spread of ARGs in the marine ecosystem and assess their impact on human health.

#### 6.1.4. Factors Affecting Antibiotic Resistance in the Aquatic Environment

**Figure 2:**
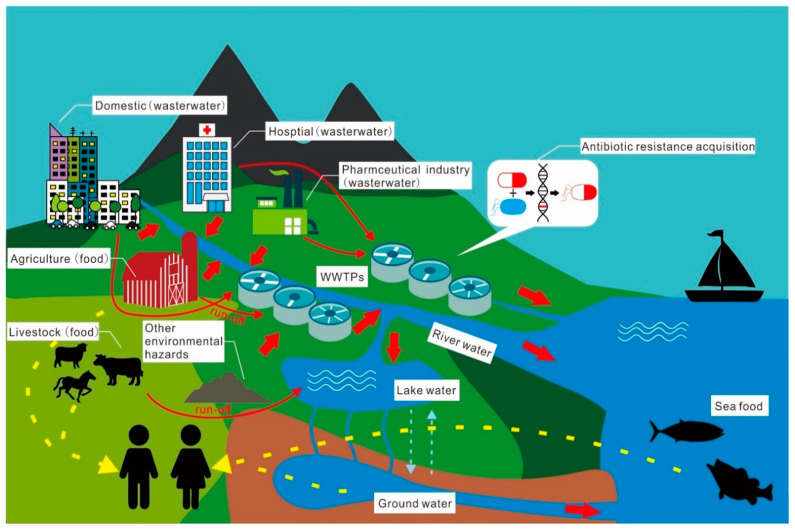
Migration, transformation, and the fate of antibiotics and their resistance in the given environment.

Antibiotic migration, transformation, and fate are analogous to and consistent with ARG transfer and accumulation in the environment (Figure 2) [189]. Antibiotics have been implicated in various studies as a critical factor in the spread of ARGs between species [190,191]. In Beijing, the *sul* gene correlated well with the concentrations of selected sulfamethazine [192]. Still, in the study by Xu et al. [190], no such correlation was found for the same set of ten sulfonamide antibiotics. Based on these results, it is possible to conclude that antibiotics are used to target the effects they have on ARGs. 

Heavy metals in the environment are also crucial in the horizontal transfer of ARGs, alongside antibiotics as the most direct source of selection pressure. Specifically, metal ions increase the permeability of cell membranes, which in turn promote the horizontal transfer of ARGs through oxidative stress, the SOS response, and the production of reactive oxygen species (ROS) [193]. There is growing evidence that the co-selection of heavy metals significantly impacts the dispersal and propagation of ARG in the natural environment. Co-selection between ARB and other pathogens could be facilitated by heavy metals used in livestock and fisheries [194]. Recently, certain metal nanoparticles (nano-alumina and Nano-TiO2) have been commonly detected as new pollutants in various environments; studies have shown that nanoparticles and heavy metals can promote the conjugate transfer of ARGs in the environment [195,196]. Other than these factors, other factors such as non-antibiotic antimicrobial chemicals, microbial community diversity, and environmental physical and chemical properties also play significant roles in AR transmission in the aquatic environment [193].

### 6.2. Terrestrial Environments

The origin of ARGs in the terrestrial environment can be traced back to spontaneous mutations and HGT, just as in the aquatic ecosystem [197]. However, the impact of antibiotics in the soil on persistence and vertical or horizontal gene transport is influenced by co-selection [197,198] and other factors such as antibiotic structure, hydrophobicity, mobility, and biodegradability [199]. It has been shown, for instance, that resident, non-pathogenic soil microbial species can better acquire ARGs in the presence of non-antibiotic stressors like heavy metals, microplastics, and pesticides [197,200,201]. This is due to a phenomenon known as cross- and co-resistance. The terms “cross-resistance” and “co-resistance” refer to two forms of resistance that can develop when the same process reduces susceptibility to antibiotics and non-antibiotics, respectively [199,202]. 

#### 6.2.1. Sludge Manure 

The sludge produced as a by-product of WWTPs can be used as a plant fertilizer or a substrate for improving soil remediation [203]. Using sewage sludge as manure is unquestionably an effective waste management strategy. Sewage sludge is rich in nutrients and organic matter, making it a good candidate for this use. However, sewage sludge harbors microorganisms resistant to multiple antibiotics, metals, plastics, and organic contaminants that pose severe threats to human and environmental health [199,204]. Recent years have witnessed a particular interest in the putative ecotoxicological effects of pharmaceuticals in the environment [198,205]. A recent assessment of treated sewage sludge used for agricultural purposes revealed the presence and accumulation of antibiotics with potential acute and short-term environmental risks [206]. In addition, compounds harmful to the environment, such as heavy metals, are frequently revealed in treated and untreated wastewater sludge [204]. In principle, antibiotics consumed by humans and animals are not entirely metabolized and are released into WWTPs as either parent or partially metabolized compounds. Together with heavy metals, residual antibiotics are not completely removed by WWTPs [207], even in advanced wastewater treatment systems that employ anaerobic digestion, coagulation, lime stabilization, membrane bioreactors, and inactivation and disinfection processes [204,208]. Therefore, if the sludge materials are deployed as manure, these compounds eventually get to the soil in concentrations relative to the treatment method and the source of waste materials. A combination of anaerobic, aerobic, and inactivation treatment strategies appears to be more effective for containing the spread of ARGs from wastewater treatment system sludge [204].

#### 6.2.2. Agricultural System

A sustainable strategy for conserving freshwater is the reuse of treated wastewater for agricultural irrigation [209]. However, wastewater irrigation is another important source of ARGs in agricultural systems. Multiple studies have shown that irrigating with wastewater increases the abundance of multidrug-resistant microorganisms and ARGs in agricultural fields [185,207,210,211]. Several ARGs conferring resistance to rifampicin, chloramphenicol, tetracycline, trimethoprim, β-lactams, aminoglycosides, fluoroquinolones, and sulfonamides, as well as tetracycline- and sulfonamide-resistant bacteria have been detected in wastewater effluent [212].

The risk of ARG spread from agricultural systems into the food chain is also of great concern. For instance, antibiotics from wastewater irrigation can accumulate in edible vegetables and, if ingested, can trigger adaptive resistance in the gut microbiome [213]. In addition, Onalenna and Rahube [214] revealed that wastewater irrigation influenced a structural shift in microbial communities and increased the spread and persistence of beta-lactamases in irrigated soil and edible vegetables. Furthermore, the distribution and persistence of ARGs from wastewater irrigation are not limited to topsoils and edible plants; the accumulation of antibiotic residues in a treated wastewater-irrigated field was found to promote the spread and persistence of ARGs among groundwater microbial communities [185]. ARG detection and spread at this depth highlight the importance of monitoring agricultural systems to control the burden of antibiotic resistance in the environment effectively.

Accordingly, one important factor determining ARG spread from wastewater irrigation is the influence of seasonal variation. Pu et al. [207] reported that the abundance of ARGs in treated wastewater was higher in summer than in winter. Based on the study of Sun et al. [215], seasonal variation impacts the nutrient content of WWTP influent, and this has a corresponding effect on the assembly mechanisms and structure of important microbial species that contribute to the removal of nutrients, pesticides, antibiotics, and other undesirable compounds during wastewater treatment. Thus, a significant change in the assembly of important microbial phylotypes due to seasonal variation reduces the efficiency of WWTPs. Overall, removing ARGs and ARB in both sludge manure and irrigation wastewater requires advancement in WWTP technologies and the development of policies that could reduce antibiotics’ misuse. This is critical because the selective pressure of residual antibiotics on microbial communities is what triggers the spread of ARGs in WWTP systems and agricultural fields.

#### 6.2.3. Manure from Livestock and Pesticides

Antibiotics are widely employed in animal breeding industries for disease prevention and treatment. However, such use could lead to the development of ABR in this sector. For example, Abdalla et al. used the farm-to-fork approach to investigate the presence of antibiotic-resistant *E. coli* in intensive pig farming in South Africa [216]. The authors analyzed 1044 pure isolates and observed an 88.5% resistance to at least one of the 20 antibiotics tested. Of greater concern in this study was that the organisms were resistant to most of the antibiotics listed in the WHO list of critically important antibiotics. Similarly, Molechan et al. used the same approach in intensive poultry farming in South Africa and found that close to 80% of all *Enterococcus* species isolated in their study were resistant to at least one of the antibiotics tested [217]. Most antibiotics used by animals (and humans) are excreted in partially metabolized or unmetabolized forms [218]. Thus, as a direct consequence, livestock manure is an important reservoir of multidrug-resistant microorganisms and ARGs. Fatoba et al. revealed that soil before chicken manure application had fewer multidrug-resistant *Enterococcus* species (10%) than soil after manure application (67.7%) [219]. However, though livestock manure is rich in nutrients and improves soil quality [220], it is also a source of undesirable materials, including antibiotics, ARG, multidrug-resistant microorganisms, and heavy metals [221]. The quantity of these materials that can be released into agricultural systems depends on the source of the livestock manure [222], the frequency of application, and soil depth [223]. For example, a recent study revealed that poultry and pig manure holds the largest reservoir of ARGs, antibiotics, and heavy metals compared to sheep and cattle manure [224]. Also, the accumulation of high-risk ARGs in topsoil depended on the frequency of manure application and the microbial communities, while the vertical migration of ARG was driven by variations in soil properties [223]. Furthermore, as with sludge manure, the spread and persistence of ARGs through livestock manure are also influenced by the co-selection of non-antibiotics like heavy metals and biocides. For example, in the co-selection of heavy metal and antibiotics resistance, Cu and Zn were the heavy metals with the strongest influence on ARG proliferation [207].

Pesticides or biocides are used to control soil-borne plant pathogens, including nematodes, oomycetes, fungi, bacteria, and other plant pathogenic groups. However, sub-lethal levels of biocides in agricultural systems impact the metabolic functionality of the soil microbiota and contribute to the evolution of microbial antibiotic resistance [225,226]. Just as with other non-antibiotic compounds like heavy metals and microplastics, pesticide stress aids the acquisition of ARGs in agricultural systems through several mechanisms, including gene mutation induction, activation of efflux pumps, and outer membrane pores inhibition [226]. For example, the co-exposure of streptomycin and pesticide in some *E. coli* strains, including O157:H7 and O103:H2, induced the emergence of significantly stronger streptomycin-resistant mutants [227]. Further, Shahid and Khan [228] demonstrated a correlation between pesticide resistance and antibiotic resistance among bacterial species recovered from the rhizosphere of edible crops. In most cases, sub-lethal pesticide levels increased the proportion of MGEs that aided the distribution of ARGs, influenced the HGT of ARGs and supported conjugation by increasing cell membrane permeability [197]. Overall, the influence of pesticides on the evolution of ARGs can be controlled by drafting policy documents for the safe use of pesticides in agricultural fields.

#### 6.2.4. Factors Affecting Antibiotic Persistence in the Terrestrial Environment

Cross- and co-resistance phenomena are important factors that determine the spread and persistence of ARGs in the terrestrial environment. Several studies have demonstrated the association between environmental ARGs and non-antibiotic compounds like heavy metals, microplastics, and pesticides [198,229,230,231]. For example, microplastics in activated sludge systems have been shown to inhibit the removal of ARGs [232] and increase microbial cooperation. Similarly, the pattern of antibiotic spread in activated sludge systems of WWTPs has been demonstrated [233]. According to the findings, stress from high concentrations of heavy metals promoted the spread of ARGs, firstly, through conjugation and subsequently through vertical gene transfer, with gram-negative bacteria detected as the highest recipients of resistant plasmids due to the selection pressure of heavy metals. Accordingly, Niu et al. [234] revealed that the passivation of heavy metals led to effective control of the abundance of bacitracin resistance genes in soil compost. Also, reducing the occurrence of cross- and co-resistance using adsorbents like biochar and clay minerals have been demonstrated as a good strategy for managing the acquisition of ARGs in the environment [235].

Other factors that influence the spread of ARGs from sludge and wastewater irrigation include the design of the WWTPs, the source of the WWTP influent, seasonal variation that impacts important microbial phylotypes, and other abiotic factors like pH and electrical conductivity [236,237]. Redesigning WWTPs to include steps that effectively remove antibiotics and other compounds of concern, like heavy metals and microplastics, could effectively reduce the spread of ARG in both wastewater sludge and effluents used for agricultural purposes, while effective policies for the administration of antibiotics in livestock farming could minimize the transfer of ARGs into agricultural systems.

### 6.3. Tools Used for Antibiotic Resistance Studies 

One of the key issues with studying resistomes and ARGs is that only a significant fraction of microbial species can be grown under laboratory conditions [238]. However, culture remains the gold standard tool for every microbiologist. Therefore, studies investigating phenotypic resistance would require culturing the microorganisms and then determining their resistance to selected antimicrobials using disk-diffusion or agar dilution methods [239]. Despite the success achieved with this approach, working with a large number of samples is challenging. Therefore, automated systems like the VITEK2 Compact system have been developed. Apart from determining the susceptibility of microorganisms, this system has the added advantage of determining the organism’s identity [240]. However, the need to culture the organisms before determining their resistance profiles limits these culture-based techniques as the resistome of a large microbial population cannot easily be determined. Therefore,, next-generation DNA sequencing (NGS) and other ‘omics’ tools are especially helpful in resistome research because some microbial species harboring ARGs are viable but non-cultivable. The *16S rRNA* gene profile PCR assay is a common method for investigating the resistome. In computational analysis, ARG profiles are linked to the microbial population using the *16S rRNA* gene as taxonomic identity markers. The use of taxonomic markers in resistome research has some drawbacks, the most significant of which is the insufficient depth of its sequences; most markers provide an accurate estimate only to the family or genus level, but not the species level [241]. Nevertheless, an integrated approach that utilizes *16S rRNA*, metagenomics, and other tools can shed light on the interactions and dynamics of the resistome in bacteria communities, whereas *16S rRNA* genes alone are limited in their ability to achieve this goal. 

Next-generation sequencing techniques like high throughput shotgun metagenomics are valuable when assessing the resistome of entire populations. This involves sequencing total genomes extracted from a population and analysing the resulting files using different bioinformatic pipelines such as PRAP (Pan Resistome Analysis Pipeline) [242]. While metagenomics is a powerful tool for investigating ARG abundance at the population level, it is more difficult because it requires significantly more computational resources than are needed to analyse the *16S rRNA* gene. Another significant restriction is that the mere presence of a gene does not ensure that it is functional or expressed by the microbial cells. Integrating metagenomics with other methods, such as functional metagenomics, could help fill this information gap [243]. Functional metagenomics does not necessitate a prior understanding of environmental ARGs. The main techniques include growth in diffusion chambers or expressing metagenomic genes in surrogate hosts for biochemical studies. These methods are not limited to any particular culture because they can also be used on non-cultivable organisms [244]. The most significant drawback is using ARGs in surrogate expression systems, which removes them from their natural environment. Functional metagenomics causes far more disruption to the natural environment than other techniques, such as *16S rRNA* gene sequencing and metagenomics [245]. Future resistome research will most likely be driven by combined functional metagenomics and culturomics techniques, particularly when used with third-generation sequencing or Nanopore sequencing techniques [243]. 

**Figure 3:**
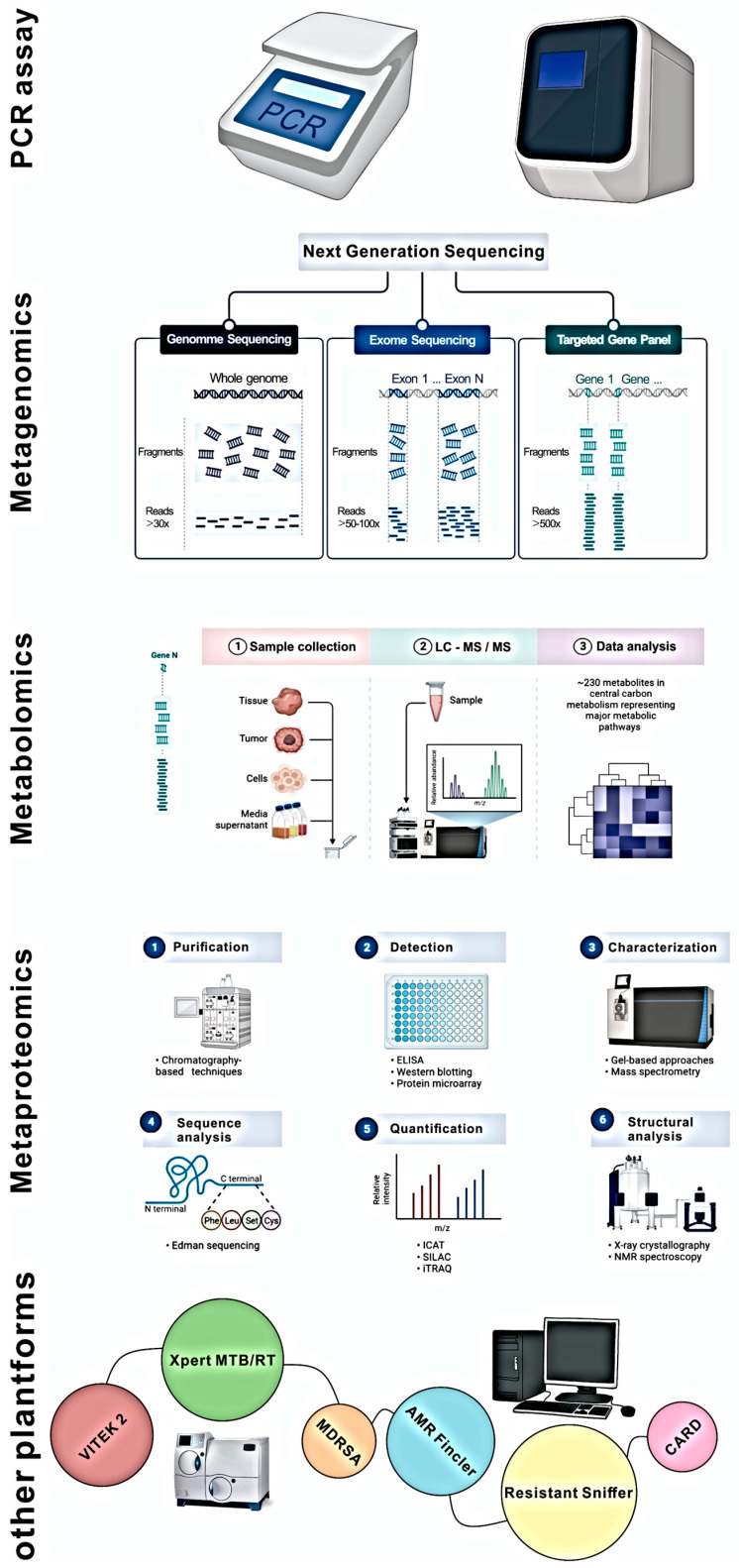
Modern tools used for resistome identification analysis.

Protein studies can offer a profound understanding of enzyme activity and the function of proteins in microbial cells that make up the resistome. Few reports on antibiotic resistance in microbial populations found in the environment have been analysed using metaproteomics or metabolomics [246]. Unlike metagenomics and metatranscriptomics, which are made easier by developments in NGS, metaproteomics and metabolomics depend on technologies that make only incremental leaps forward. These technologies include two-dimensional or differential in-gel electrophoresis, liquid chromatography with mass spectrometry, or MALDI-TOF [247,248]. To make these methods applicable to future resistome studies, there is a need for advancements in high throughput sampling techniques. Using metaproteomics and metabolomics in resistome research can answer questions about the mechanisms involved in developing resistomes, although the scope of this research is limited. Figure 3 depicts modern techniques used in resistome studies.

### 6.4. Ecotoxicological Impact of Antibiotics and Antibiotic-Resistant Bacteria

The persistence of antibiotics in the environment can alter the ecosystem’s community structure and ecological function, including biomass and biodiversity, as well as the survival, reproduction, metabolism and population of organisms [249]. Multiple investigations have demonstrated that antibiotics may have physiological effects on non-target organisms like plants and other living organisms. For instance, many antibiotic classes have been found to share common receptors in plants; these include those that inhibit chloroplast replication (fluoroquinolones), transcription and translation (tetracyclines, macrolides, lincosamides, P-aminoglycosides, and pleuromutilins), metabolic pathways (folate biosynthesis, *sul*fonamides, and triclosan), and sterol biosynthesis (triclosan and other classes of statin-type blood lipid regulators) [250]. Similarly, numerous studies have evaluated the effects of antibiotics on non-target sensitive organisms like zebrafish, Daphnia, algae, mussels, and other aquatic organisms and have reported general toxicity indicators like LC_50_, EC_50_, and mean inhibitory concentration (IC_50_) [251]. However, existing standard ecotoxicology tests used in the regulatory assessment of pharmaceuticals have been questioned due to possible inadequacies in capturing ecologically significant effects [252]. Due to a lack of information, a thorough analysis of environmental risks cannot be conducted at this time. There is still a dearth of primary data on antibiotics and ARGs’ environmental fate and impacts. Such information must be readily available to conduct accurate risk assessments and implement effective risk management programs.

## 7. Antibiotic-Resistant Bacteria and Human Health Concerns

Antibiotic resistance or drug resistance is a worldwide public health crisis requiring immediate action. In 2019, antimicrobial resistance was linked to 4.95 million deaths, 1.27 million of which were attributed to drug-resistant illnesses alone. Without concerted action, this number might exceed ten million by 2050, costing more than USD 100 trillion [253]. In recent decades, many pathogenic bacteria have evolved into multi-drug resistant (MDR) bacteria. For instance, the US Centre for Disease Control and Prevention (CDC) released a list of the top public threats in 2015, which included drug-resistant diseases and classified them as Urgent, Serious, or concerning threats (Table 4). Four urgent threats include carbapenem-resistant *Enterobacteriaceae* and *Acinetobacter*, drug-resistant *N. gonorrhoeae* and *Clostridium difficile*, causing numerous deaths annually in the US and other countries. Serious infections include those indicated in Table 4, such as methicillin-resistant *S. aureus* (MRSA) and *van*comycin-resistant *Enterococcus* (VRE) infections and extremely drug-resistant tuberculosis (XDR-TB). Other members of this category include *S. pneumoniae*, which accounts for most bacterial pneumonia and meningitis worldwide, and *Acinetobacter*, *Campylobacter*, fluconazole-resistant *Candida*, *Enterobacteriaceae* (*Pseudomonas aeruginosa*, *Salmonella* (both typhi and non-typhi) generating a beta-lactamase that has extensive activity against most penicillins and cephalosporins. It is estimated that this group is responsible for around 22,500 deaths annually in the United States. In addition, the presence of streptococci resistant to erythromycin and clindamycin is viewed as “of concern” [16]. While these concerns focus more on human consumption of antibiotics, it should be noted that these problematic organisms could also originate from animal sources—directly through contact with infected animals or indirectly through the consumption of contaminated animal products. For example, hospitalized pets have been recognized as significant reservoirs and sources of carbapenem-resistant bacteria, including *Acinetobacter* spp. [254], implying a possible direct transmission from these animals to humans. These carbapenem-resistant bacteria have also been identified in food animals [255,256], meaning that indirect transmission to humans could occur through the consumption of these animals as a protein source.

## 8. Strategies for Addressing the Challenge of Antibiotic Resistance

The challenge posed by antibiotic-resistant bacteria can be categorized into five primary intervention strategies within the human and veterinary sectors. In the first place, infection prevention and control principles continue to be the cornerstone in the fight against the spread of ABR [22]. Second, vaccinations are a critical tool for preventing infections and decreasing the demand for antibiotics. Although new vaccine initiatives are being developed for *S. aureus*, *E. coli*, and others, vaccines are only available for one of the six leading pathogens (*S. pneumoniae*) [257]. Third, reducing exposure to antibiotics for purposes other than treating human disease is an essential potential risk-reduction strategy. An increase in ABR in humans has been linked to the widespread use of antibiotics in agriculture, though the exact cause-and-effect relationship is still up for debate [258]. Intensive farming imposes stress on food animals, forcing farmers to use antibiotics to treat their animals [259]. However, treating individual sick animals is challenging; hence, providing antibiotics to all animals on a farm prophylactically through their feed and water helps reduce the disease burden and improves animal health. This is not without consequences, as ABR is favoured under such conditions. Combating this phenomenon would require the observation of stringent biosecurity measures [260] and the use of alternatives to antimicrobials to treat sick animals [261]. Fourth, antibiotics should not be used to treat viral infections unless necessary. For antimicrobial use can be reduced or stopped when necessary, it is critical to establish mechanisms that facilitate rapid and accurate diagnosis of disease by clinicians [262]. Finally, it is essential to continue investing in the pipeline for developing new antibiotics and providing access to second-line antibiotics in areas that do not have widespread access [263]. It is an urgent priority to identify strategies that can reduce the burden of bacterial ABR across a wide range of settings or specifically tailored to the available resources and the leading pathogen–drug combinations in a particular setting.

The environmental dimension of ABR remains the least addressed component in the fight against this global ill. This is partly because the environment presents a more complex scenario involving numerous stressors than humans and animals. Nevertheless, wastewater treatment plants have been recognized as hotspots for the dissemination of ABR in the environment. Therefore, improving the quality of the effluents from these plants would reduce the discharge of polluted waters containing antimicrobial-resistant pathogens and ARGs into receiving water bodies. This is, however, challenging for areas in low- and middle-income countries where such facilities are unavailable. Nevertheless, greater sensitization and the provision of mobile toilets could prevent open defecation and pollution of the environment.

Although these strategies have been presented separately, it must be noted that for effective, sustainable solutions to be achieved in the fight against ABR, firm collaborations and communication must be established between actors in the One Health triad—humans, animals, and the environment.

## 9. Conclusions

Antibiotic resistance remains a significant challenge threatening human, animal and environmental health. Although ABR has increased over the years due to the indiscriminate use of antibiotics in human and veterinary settings, ABR is also shown to be a natural process, with resistance genes discovered in pristine environments with little or no human interference. Furthermore, although less studied, the environmental dimension of ABMR constitutes a significant reservoir as a source of ABR through horizontal and vertical transfer, with plasmids and other mobile genetic elements playing a crucial role in this process. Within the environment, other less-considered factors like heavy metals and pesticides also play an important role in selection pressure, inducing resistance in previously susceptible environmental organisms. Furthermore, wastewater treatment plants remain major contributors of ARB and ARGs in the environment. Given the broad distribution of ABR, solutions aiming to curb this ill should be multifacet, involving antimicrobial stewardship in humans and animals, prevention of environmental pollution and promoting the discovery of new antibiotics, among others.

## Figures and Tables

**Table 1:** Natural and Synthetic antimicrobials from 1910–2010 [2,7].

Year of Discovery	Microorganisms			Synthetic Antimicrobials
	Actinomycetes	Bacteria	Fungi	
1910–1940				Salvarsan Sulfonamides sufapyridine
1940	Streptomycin Aminoglycosides Tetrecyclines Amphenicols	Polypeptides Bacitracin	Penicillins	Sulfones Salicylates
1950	Macrolides Glycopeptides Tuberactinomycins	Polymyxins		Nitrofurans Pyridinamides
1960	Ansamycins Lincosamides Streptogramins Cycloserine		Fusidic acid Cephalosporins Enniatins	Quinolones Azoles Thioamides Ethambutol Phenazines Diaminopyrimidines
1970	Phosphonates Fosfomycin			
1980	Carbapenems	Mupirocin Monobactams		
2000	Lipopetides		Pleuromutilins	Oxazolidinones linezolid
2010	Liparmycins			Diarylquinolines

**Table 3:** Different mechanisms of antibiotic resistance with examples.

Antimicrobial Agents	Mechanism of Action	Examples	References
Penicillins and cephalosporins	Enzymatic inactivation by β-lactamase; enzymatic modification by acylase and esterase; outer membrane protein deletion; alteration of penicillin-binding proteins	β-lactamase containing gram-negative rods	[117,118,119]
Monobactams	Enzymatic inactivation by β-lactamase	*Haemophilus influenza*; *Pseudomonas aeruginosa*	[120,121]
Carbapenems	Enzymatic inactivation by β-lactamase; outer-membrane protein deletion	*Neisseria gonorrhoea*; *A. baumannii*; *Citrobacter portucalensis*; *K. pneumoniae*; *Escherichia coli*	[122,123]
Carbacephems	Cell wall synthesis inhibition	*ESBL-producing Enterobacteria*	[124]
Imipenem	Decreased Cell membrane permeability	*Pseudomonas* sp.; *K.*	[125,126]
*Van*comycin	Inhibition of glycopeptides access	*S. aureus*; *Enterococcus* sp.	[127,128]
Trimethoprim	Increased production of dihydrofolate reductase; production of trimethoprim-insensitive dihydrofolate reductase	*Streptococcus agalactiae*; *E. coli*; *Burkholderia pseudomallei*	[129,130,131]
*Sul*fonamides	Increased production of p-aminobenzoic acid; increased production of pteridine; increased production of sulfonamide-insensitive dihydropteroate synthetase	*Haemophilus influenza*; *S. pneumoniae*; *S. pyogenes*; *Neisseria meningitidis*	[132]
Aminoglycosides	Enzymatic modification by acetylation, phosphorylation, and nucleotidylation; ribosomal alteration; diminished drug uptake	*Clostridium perfringens*; *Bacteroides fragilis*; *S. aureus*; *Bacillus cereus*	[133,134,135]
Chloramphenicol	Enzymatic inactivation by acetylation; decreased drug permeability	*Streptomyces venezuelae*; *Pseudomonas putida*; *Pneumococcus* sp.; *E. coli*	[136,137,138,139]
Macrolides	Enzymatic modification by esterase; alteration of 23S ribosomal RNA	*S. pneumoniae*, *S. aureus*	[137,140]
Lincosamides	Enzymatic modification by nucleotidylation or phosphorylation; alteration of 23S ribosomal RNA	*S. pneumoniae*; *S. agalactiae*; *Acinetobacter baumannii*	[140,141,142]
Tetracyclines	Active efflux preceded by chemical modification; ribosomal alterations	*E. coli*, *Shigella* sp., *S. pneumoniae*, *S.s aureus*, *Clostridium perfringens*, *Helicobacter pylori*	[143,144]
Quinolones	Alteration of subunit A of DNA gyrase; decreased drug permeability	*Stenotrophomonas maltophilia*; *Pseudomonas* species; *Enterobacteriaceae*	[145,146,147]

**Table 4:** Public threat status of different antimicrobial-resistant organisms.

Threat Status	Organism	Estimated Clinical Cases Per Year	Estimated Healthcare Cost (US Dollars)	Descriptions
Urgent	Carbapenem-resistant *Acinetobacter*	8500 (700)	281 million	Carbapenem-resistant *Acinetobacter* causes pneumonia and wound, bloodstream, and urinary tract infections. Nearly all these infections happen in patients who recently received care in a healthcare facility
	*Clostridioides difficile* (*C. difficile*)	223,900 (12,800)	1 billion	*C. difficile* causes life-threatening diarrhoea and colitis (inflammation of the colon), mostly in people who have had both recent medical care and antibiotics.
	Carbapenem-resistant *Enterobacterales* (CRE)	13,100 (1100)	130 million	CRE are a major concern for patients in healthcare facilities. Some *Enterobacterales* are resistant to nearly all antibiotics, leaving more toxic or less effective treatment options.
	Drug-resistant *N. gonorrhoeae* (*N. gonorrhoeae*)	550,000	133.4 million	*N. gonorrhoeae* causes the sexually transmitted disease *Gonorrhoea* that can re*sul*t in life-threatening ectopic pregnancy and infertility and can increase the risk of getting and giving HIV.
Serious	Drug-resistant *Campylobacter*	448,400 (70)	270 million	*Campylobacter* usually causes diarrhoea (often bloody), fever, and abdominal cramps and can spread from animals to people through contaminated food, especially raw or undercooked chicken
	ESBL-producing *Enterobacterales*	197,400 (9100)	1.2 billion	ESBL-producing *Enterobacterales* are a concern in healthcare settings and the community. They can spread rapidly and cause or complicate infections in healthy people. ESBLs are enzymes that break down commonly used antibiotics, such as penicillins and cephalosporins, making them ineffective.
	*Van*comycin-resistant *Enterococcus* (VRE)	54,500 (5400)	539 million	*Enterococci* can cause severe infections for patients in healthcare settings, including bloodstream, surgical site, and urinary tract infections.
	Multidrug-resistant *Pseudomonas aeruginosa* (*P. aeruginosa*)	32,600 (2700)	767 million	*P. aeruginosa* infections usually occur in people with weakened immune systems and can be particularly dangerous for patients with chronic lung diseases.
	Drug-resistant non-typhoidal *Salmonella*	212,500 (70)	400 million	Non-typhoidal *Salmonella* can spread from animals to people through food and usually causes diarrhoea, fever, and abdominal cramps. Some infections spread to the blood and can have life-threatening complications.
	Drug-resistant *Salmonella* serotype Typhi	4100 (<5)	11 to 21 million	*Salmonella* Typhi causes severe typhoid fever, which can be life-threatening. Most people in the U.S. become infected while traveling to countries where the disease is common.
	Drug-resistant *Shigella*	77,000 (<5)	93 million	*Shigella* spreads in feces through direct contact or contaminated surfaces, food, or water. Most people with *Shigella* infections develop diarrhoea, fever, and stomach cramps.
	Methicillin-resistant *S. aureus* (MRSA)	323,700 (10,600)	1.7 billion	*S. aureus* are common bacteria that spread in healthcare facilities and the community. In addition, MRSA can cause difficult-to-treat staph infections because of resistance to some antibiotics.
	Drug-resistant *S. pneumoniae*	900,000 (3600)	4 billion	*S. pneumoniae* causes pneumococcal disease, ranging from ear and sinus infections to pneumonia and bloodstream infections
	Drug-resistant Tuberculosis	847 (62)	1.6 million	TB is caused by *M. tuberculosis*. It is among the most common infectious diseases and a frequent cause of death worldwide.
Concerning	Erythromycin-resistant Group A *Streptococcus (GAS)*	5400 (450)	2.6 million	GAS can cause many infections ranging from minor illnesses to severe and deadly diseases, including strep throat, pneumonia, flesh-eating infections, and sepsis.
	Clindamycin-resistant Group B *Streptococcus (GBS)*	13,000 (720)	NA	GBS can cause severe illness in people of all ages.
Watch list	Azole-resistant *Aspergillus fumigatus*	NA	NA	*Aspergillus fumigatus*, a ubiquitously distributed opportunistic pathogen, is the leading agent of aspergillosis, ranking first among fungal killers.
	Drug-resistant *Mycoplasma genitalium*			*Mycoplasma genitalium* is one of the important causes of non-gonococcal urethritis.
	Drug-resistant *Bordetella perstussis*			Pertussis (whooping cough), a highly contagious respiratory illness caused by *Bordetella pertussis*

## Data Availability

All articles reviewed have been included in the reference list.
